# Association between Unplanned Conversion and Patient Survival after Laparoscopic Liver Resection for Hepatocellular Carcinoma: A Propensity Score Matched Analysis

**DOI:** 10.3390/jcm13041116

**Published:** 2024-02-16

**Authors:** Boram Lee, Jai Young Cho, Ho-Seong Han, Yoo-Seok Yoon, Hae Won Lee, MeeYoung Kang, Yeshong Park, Jinju Kim

**Affiliations:** Department of Surgery, Seoul National University Bundang Hospital, Seoul National University College of Medicine, Gumi-ro, 173, Bundang-gu, Seongnam-si 13620, Gyeonggi-do, Republic of Korea; boramlee0827@snubh.org (B.L.); hanhs@snubh.org (H.-S.H.); yoonys@snubh.org (Y.-S.Y.); 82637@snubh.org (M.K.); 82750@snubh.org (J.K.)

**Keywords:** laparoscopy, conversion to open surgery, hepatectomy, carcinoma, hepatocellular, survival

## Abstract

Unplanned conversion (UPC) is considered to be a predictor of poor postoperative outcomes. However, the effects of UPC on the survival of patients with hepatocellular carcinoma (HCC) remain controversial. The aim of this study is to compare the outcomes between patients who underwent laparoscopic liver resection (LLR) and those who underwent UPC for HCC. Among 1029 patients with HCC who underwent hepatectomy between 2004 and 2021, 251 were eligible for the study. Of 251 patients who underwent hepatectomy for HCC in PS segments, 29 (26.0%) required UPC, and 222 underwent LLR. After 1:5 PSM, 25 patients were selected for the UPC group and 125 for the LLR group. Blood loss, transfusion rate, hospital stay, and postoperative complication were higher in the UPC group. Regarding oncologic outcomes, although the 5-year overall survival rate was similar in both groups (*p* = 0.544), the recurrence-free survival rate was lower in the UPC group (*p* < 0.001). UPC was associated with poor short-term as well as inferior long-term outcomes compared with LLR for HCC in PS segments. Therefore, surgeons must carefully select patients and consider early conversion if unexpected bleeding occurs to maintain safety and oncologic outcomes.

## 1. Introduction

Laparoscopic liver resection (LLR) is now widely accepted as a treatment option for hepatocellular carcinoma (HCC) [1]. With advances in surgical instruments and accumulated experience in performing laparoscopic surgery, LLR has demonstrated acceptable oncologic outcomes for minor and major liver resection relative to open liver resection (OLR) [2]. In addition, LLR was superior to OLR in terms of short-term outcomes such as postoperative complications, blood loss, postoperative hospital stay, and functional recovery [3]. LLR has proven to be a safe and feasible treatment option for HCC and is now considered a standard procedure for HCC resection in many centers worldwide. However, these positive outcomes are obtained when LLR is safely completed.

Conversion to open surgery might nullify the benefits of laparoscopic surgery. Emergency conversion was associated with significant increases in postoperative complication rates and length of stay and, more importantly, higher 30- and 90-day mortality rates [4,5,6]. Because most of the indications for emergency conversion were related to bleeding or damage to the surrounding structures, conversion is associated with poor short-term outcomes after surgery. Nevertheless, the impact of conversion to open surgery on long-term outcomes remains controversial. Several studies have demonstrated that conversion to open surgery may be associated with adverse long-term oncologic outcomes in laparoscopic colorectal surgery [7,8,9]. However, other studies have reported similar oncological outcomes after colectomy between converted and non-converted patients [10,11]. To date, few studies have investigated the clinical impact of conversion to open surgery compared with LLR.

LLR in the posterosuperior (PS) segments (Segments 1, 4A, 7, and 8) is a technically challenging procedure because of the difficulty of exposing deeply located lesions, isolating the target Glissonean pedicle, and accurately determining the cutting plane during parenchymal transection [12,13,14]. For these reasons, a multivariable analysis revealed that lesions located in the PS segments are a predictive risk factor for the conversion to open surgery irrespective of the learning curve for LLR [15]. Therefore, in this study, we compared the surgical and oncological outcomes between patients who required unplanned conversion during LLR with those of patients who underwent successful LLR for HCC located in PS segments after matching the groups using the propensity score matching (PSM) method.

## 2. Materials and Methods

This retrospective study was approved by the institutional review board of Seoul National University Bundang Hospital, Seongnam, Republic of Korea, which is an academic hospital affiliated with Seoul National University, College of Medicine (B-2212-799-102). The medical records of consecutive patients who underwent liver resection for HCC between January 2004 and May 2021 were retrieved from the institution’s prospectively collected databases.

Among 1029 patients identified from the databases, we reviewed the records for 298 consecutive patients who underwent curative intent LLR for HCC in the PS segments by the same surgical team. LLR was performed by four surgeons, all of whom had more than 100 cases of experience. After applying the exclusion criteria (conversion to open surgery owing to advanced disease, *n* = 21; conversion owing to severe adhesion, *n* = 12; and patients with incomplete data, *n* = 14), 251 patients were included in this study. Of these, 222 underwent LLR, and 29 required UPC owing to massive bleeding during surgery.

As part of preoperative planning for liver resection, the surgeon determined whether to use an open and laparoscopic approach based on the tumor size, tumor location, and hepatic function. In general, LLR was performed for tumors located ≥5 cm from the major vascular or biliary structures, allowing the surgeon to technically secure the surgical margin. Absolute contraindications to LLR included the need for vascular resection and reconstruction or en-bloc multi-visceral resection. All of the patients were discussed in multidisciplinary meetings to assess the feasibility of the planned surgical approach. For this study, we defined UPC as attempted LLR, which required unscheduled conversion to open surgery owing to bleeding.

### 2.1. Variables

Data collected from the medical records included patient demographics, preoperative disease characteristics, operative details, pathological outcomes, and survival data. We used 1:5 PSM to limit selection bias and to match patients based on preoperative clinical factors. After adjusting for these factors, the short- and long-term operative outcomes were compared between the two matched groups (LLR and UPC groups). The extent of hepatectomy was classified according to the Brisbane 2000 terminology [16]. Postoperative complications (within 30 days after surgery) were based on the most severe complication and were graded according to the Clavien-Dindo (C-D) classification [17]. Major complications were defined as those with a C-D grade of ≥III.

### 2.2. Survival Outcomes

Patients were followed up by abdominal computed tomography and blood tests, including measurement of tumor markers, every 3 months for the first 2 years after primary surgery and then every 3–6 months thereafter. Overall survival (OS) was defined as the time from primary surgery to the date of death, regardless of cause. Recurrence-free survival (RFS) was defined as the time from primary surgery to the first documented detection of recurrence during regular follow-up.

### 2.3. Statistical Analysis

Statistical analyses were performed using SPSS software package version 25 for Windows (IBM Corporation, Armonk, NY, USA). The demographic, perioperative, and clinical data were summarized using descriptive statistics and are presented as the mean ± standard deviation unless otherwise stated. Student’s t-test or the Mann-Whitney U test was used to compare continuous variables, and Pearson’s chi-squared test was used to compare categorical variables. OS and RFS were evaluated using Kaplan-Meier curves. To identify variables that were independently associated with UPC, we performed logistic regression analysis with backward stepwise variable selection at a significance level of *p* < 0.05. We also performed Cox proportional hazards regression modeling to examine the strength of the association between covariates and survival times. All analyses were performed using a two-tailed α-value of 0.05, and *p* < 0.05 or the 95% confidence interval (CI) indicated statistical significance.

## 3. Results

Among 251 patients who underwent liver resection for HCC located in PS segments, 29 (26.0%) required UPC, and 222 underwent LLR. After 1:5 PSM, 25 patients were selected for the UPC group, and 120 were selected for the LLR group.

### 3.1. Demographic and Disease Characteristics

Table 1 shows the patients’ demographic and disease characteristics, as well as both groups matched by PSM. Before PSM, the proportion of patients with a Child-Pugh score of B was significantly greater in the UPC group (17.2% vs. 1.8%, *p* = 0.001), and mean tumor size was significantly larger in the UPC group than in the LLR group (4.2 ± 2.8 vs. 3.3 ± 2.2 cm, *p* = 0.013). After PSM, the patient characteristics were well balanced in both groups, with no significant differences in demographic variables (age, body mass index (BMI), sex, hypertension, diabetes, and prior abdominal surgery), liver-related factors (virology, model for end-stage liver disease score, bilirubin, alanine aminotransferase, aspartate aminotransferase, international normalized ratio, platelet count, and Child-Pugh score), and tumor-related factors (prior transarterial chemoembolization, radiofrequency ablation, preoperative α-fetoprotein level, and mean tumor size).

### 3.2. Surgical and Oncological Outcomes

Table 2 shows the surgical and oncological outcomes in both groups after PSM. The proportion of patients who underwent tumorectomy was similar in the UPC and LLR groups (44.0% vs. 43.2%). Although the operation time was not significantly different between the UPC and LLR groups (337.2 ± 203.1 vs. 302.7 ± 173.2 min, *p* = 0.254), blood loss (3172.1 ± 4527.0 vs. 809.4 ± 1026.4 mL, *p* < 0.001) and the intraoperative blood transfusion rate (48.0% vs. 24.8%, *p* = 0.028) were both significantly greater in the UPC group. The major complication rate (28.0% vs. 18.1%, *p* = 0.042) and the length of hospital stay (14.8 ± 18.3 vs. 8.9 ± 8.3 days, *p* = 0.015) were also significantly greater in the UPC group. There were no differences between the two groups with regard to pathologic characteristics (R0 resection rate, surgical margin, microvascular invasion, serosal invasion, and tumor stage).

### 3.3. Survival Analysis

**Figure 1:**
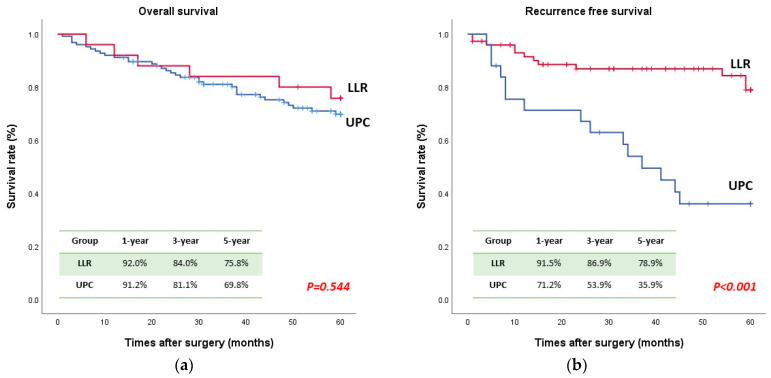
Survival curve ((**a**) overall survival; (**b**) recurrence-free survival).

The median follow-up of the whole study population was 40 months (range 1–203 months). The OS and RFS curves are displayed in Figure 1. The OS curves were similar in both groups; the 1-, 3-, and 5-year OS rates were 91.2%, 81.1%, and 69.8%, respectively, in the UPC group and 92.0%, 84.0%, and 75.8%, respectively, in the LLR group (*p* = 0.544). However, the 1-, 3-, and 5-year RFS rates were significantly worse in the UPC group (71.2%, 53.9%, and 35.9%, respectively) than in the LLR group (91.5%, 86.9%, and 78.9%, respectively; *p* < 0.001).

### 3.4. Univariate and Multivariable Analysis of Risk Factors Associated with Poor OS and RFS

Table 3 presents the factors associated with poor OS and RFS after liver resection. The preoperative BMI < 18.5 kg/m^2^, preoperative hypoalbuminemia (<3.5 g/dL), presence of cirrhosis, tumor size > 5 cm, operation time > 300 min, intraoperative blood loss > 500 mL, intraoperative transfusion, microvascular invasion, pathologic T stage, and hospital stay >7 days were associated with poor OS in the univariate analyses. In the multivariable analysis, preoperative BMI < 18.5 kg/m^2^ (hazard ratio [HR] 2.073; 95% CI 1.055–4.072; *p* = 0.034), hypoalbuminemia (HR 3.497; 95% CI 1.600–7.464, *p* = 0.002), operation time > 300 min (HR 2.840; 95% CI 1.121–7.194, *p* = 0.028), microvascular invasion (HR 2.503; 95% CI 1.022–6.708, *p* = 0.042), and pT4 (HR 6.692; 95% CI 1.119–14.698, *p* = 0.041) were associated with poor OS. The operation type (LLR vs. UPC) was not significantly associated with OS.

For RFS, preoperative thrombocytopenia (<100 × 10^3^/µL), operation type, intraoperative blood loss > 500 mL, intraoperative transfusion, and microvascular invasion were significantly associated with poor RFS. In the multivariable analysis, preoperative thrombocytopenia (HR 2.081; 95% CI 1.161–3.370, *p* = 0.014), intraoperative blood loss > 500 mL (HR 2.194; 95% CI 1.119–4.299; *p* = 0.022), and microvascular invasion (HR 2.401; 95% CI 1.344–4.288, *p* = 0.003) were significantly associated with poor RFS. Furthermore, UPC was significantly associated with worse RFS (HR 2.203; 95% CI 1.045–4.643; *p* = 0.038).

### 3.5. Risk Factors Associated with UPC

Table 4 shows the results of the univariate and multivariable logistic regression analyses of preoperative variables associated with UPC. In univariable analysis, potential risk factors for conversion included hypoalbuminemia and tumor size ≥ 3 cm. In the multivariable analysis, only hypoalbuminemia was significantly associated with an increased likelihood of UPC (HR 4.873, 95% CI 1.904–12.471, *p* = 0.001).

## 4. Discussion

This is the first study to assess the oncologic outcomes of UPC in patients undergoing LLR for HCC located in the PS segments. In this study, UPC was associated with poor short-term outcomes as well as inferior RFS compared with LLR. Moreover, UPC was an independent predictive factor for poor RFS.

Minimally invasive surgery (MIS) has notable benefits in terms of reduced postoperative pain, shorter hospital stays, and faster recovery time compared with conventional surgery. Among the types of MIS for various organs, LLR is a procedure that can maximize the advantages of MIS because it does not require anastomosis or vascular resection [18]. Indeed, several systemic reviews and meta-analyses comparing surgical types (LLR vs. OLR) have shown that LLR has short-term clinical advantages and comparable long-term oncologic outcomes [19,20,21]. However, a double-edged sword of MIS is that the risk of unexpected conversion to open surgery should always be taken into account. Conversion to open surgery may not only reduce or eliminate the advantages associated with laparoscopic surgery but may even result in a worse prognosis than would be expected.

In this study, the surgical outcomes following UPC included greater intraoperative blood loss, greater transfusion requirements, longer hospital stay, and increased morbidity compared with LLR, consistent with the results of previous studies [9,21,22,23]. These results are somewhat predictable because the most common reason for UPC is uncontrolled bleeding during LLR. It is also well known that excessive blood loss is an important predictor of postoperative complications after hepatectomy [23,24].

However, the clinical significance of UPC regarding long-term oncologic outcomes is controversial. Stiles et al. reported that UPC was associated with poor OS compared with successful LLR and more predominant differences in major resection [25]. By contrast, Lee et al. demonstrated no statistically significant difference in OS between LLR and open conversion [22]. They explained that these results were due to early conversion after considering the operation time and surgical difficulty. It is important to remember that the clinical significance of UPC may vary according to the clinical situation and the timing of conversion. Therefore, in this study, we performed an analysis of patients who required UPC owing to unexpected massive bleeding.

Although the OS was similar between the two groups, RFS was worse in the UPC group than in the LLR group in our study. Notably, UPC was an independent risk factor for poor RFS. There are several factors that may explain this adverse effect of UPC on survival. First, the excessive blood loss and transfusion in the UPC group may introduce an immunologic disadvantage compared with the LLR group. In previous studies, excessive blood loss during liver resection was significantly associated with poor oncologic outcomes [26,27,28]. One possible explanation is that a hypoxic environment could promote the epithelial-mesenchymal transition, which makes the residual tumor cells more aggressive [29]. In addition, in transfused patients, the most reasonable explanation for the worse recurrence rate in patients with HCC is the immunosuppressive effect on the host that occurs after transfusion, a process termed transfusion-related immunomodulation [30,31]. Second, in this study, the rate of major complications was significantly greater in the UPC group than in the LLR group. Chok et al. reported that postoperative complications could lead to adverse long-term outcomes after resection of HCC [32]. Zhou et al. reported that the occurrence of postoperative complications is a predictive factor for HCC recurrence, especially early recurrence, after curative hepatectomy [33]. One possible factor that promotes metastatic growth and early recurrence is immunosuppression resulting from systemic inflammatory responses [34]. Third, remnant liver ischemia (RLI) in the UPC group may be associated with poor oncologic outcomes. Cho et al. reported that longer operative time was an independent risk factor for severe RLI after hepatectomy [34]. Because patients in the UPC group had a longer operative time and suffered hypoxic damage owing to excessive bleeding, it seems likely that they developed RLI. Patients with severe RLI had a higher recurrence rate and a lower RFS rate after hepatectomy compared with patients with minimal RLI [35]. Although the mechanisms underlying the worse prognosis of patients with severe RLI are poorly understood, liver ischemic injury can lead to lymphocyte dysfunction and promote the release of cytokines and chemokines [36]. If this period of suppressed immunity is prolonged by severe RLI, it may lead to increased growth of occult micrometastases [35]. For these reasons, the poor long-term outcomes of UPC for hepatectomy seem inevitable.

Careful patient selection and a step-by-step approach are recommended to ensure the patient’s safety when considering LLR. Moreover, as the subject of this study, LLR for a tumor located in the PS segments is technically difficult, requiring very strict patient selection. Troisi et al. reported that, according to the surgeon’s experience and irrespective of the learning curve, resection of the PS segments was identified as an independent risk factor for the conversion of LLR [15]. In this study, hypoalbuminemia was significantly associated with an increased likelihood of conversion. Although our study did not demonstrate a significant impact of the cirrhosis on conversion, it is important to acknowledge the centrality of cirrhosis and portal hypertension (PTN) in the laparoscopic liver resection [37].

This study has several limitations. First, although this study included patients who required UPC owing to bleeding, we cannot exclude potential selection bias due to the retrospective design of the study. However, by prioritizing uncontrolled bleeding as a key inclusion criterion, we aim to focus on factors that have a more direct and clinical impact on patient outcomes. Second, the small sample size may reduce the power of the study and increase the margin of error. Therefore, further prospective registry data are required to gather more meaningful findings. Lastly, since our study predominantly included cases performed after the learning curve for LLR, it was influenced to a lesser extent by the surgeon’s experience. However, surgeon experience is a significant prognostic factor in LLR and remains an important consideration. Despite these limitations, this is the first study to analyze the significance of UPC when performing LLR for tumors located in the PS segments.

## 5. Conclusions

In conclusion, the present study showed that UPC in patients undergoing LLR for HCC located in PS segments was associated with poor short-term outcomes, as well as inferior RFS, compared with LLR. Therefore, patient selection and early conversion in the event of unexpected bleeding should be carefully considered to ensure the patient’s safety and oncologic outcomes. Further prospective studies are required to confirm the impact of UPC on patient outcomes.

## Figures and Tables

**Table 1:** Baseline patient and disease characteristics.

	Unmatched			After Applying PSM		
Variables	UPC (*N* = 29)	LLR (*N* = 222)	*p* Value	UPC (*N* = 25)	LLR (*N* = 120)	*p* Value
**Demographic data**						
Age (years)	62.1 ± 10.5	61.2 ± 10.1	0.569	61.9 ± 10.5	61.4 ± 10.2	0.742
Male (*n* [%])	22 (75.9%)	171 (77.0%)	0.820	18 (72.0%)	94 (75.2%)	0.802
BMI (kg/m^2^)	24.8 ± 3.2	24.8 ± 3.3	0.929	24.8 ± 3.4	24.6 ± 3.1	0.659
HTN (*n* [%])	14 (48.3%)	101 (45.5%)	0.844	13 (52.0%)	57 (45.6%)	0.662
Diabetes (*n* [%])	10 (34.5%)	65 (29.3%)	0.666	9 (36.0%)	38 (30.4%)	0.639
Prior abdominal surgery (*n* [%])	6 (20.7%)	70 (31.5%)	0.286	6 (24.0%)	29 (23.2%)	1.000
**Preoperative data**						
Etiology (*n* [%])			0.451			0.607
Hepatitis B	16 (55.2%)	148 (67.0%)		14 (56.0%)	82 (66.1%)	
Hepatitis C	2 (6.9%)	12 (5.4%)		2 (8.0%)	9 (7.3%)	
MELD score	7.9 ± 1.3	7.7 ± 2.1	0.590	7.9 ± 1.2	7.9 ± 2.4	0.550
Child-Pugh score			0.001			0.262
A	24 (82.8%)	218 (98.2%)		23 (92.0%)	121 (96.8%)	
B	5 (17.2%)	4 (1.8%)		2 (8.0%)	4 (3.2%)	
Bilirubin (mg/dL)	0.8 ± 0.5	0.8 ± 0.4	0.695	0.7 ± 0.5	0.8 ± 0.4	0.477
ALT (IU/L)	43.4 ± 26.6	46.5 ± 83.1	0.524	43.9 ± 28.3	49.8 ± 102.8	0.503
AST (IU/L)	49.4 ± 29.2	48.7 ± 87.3	0.725	50.1 ± 31.2	55.2 ± 111.7	0.513
Albumin (g/dL)	3.9 ± 0.6	4.2 ± 0.4	0.003	4.0 ± 0.5	4.1 ± 0.5	0.976
INR	1.1 ± 0.1	1.1 ± 0.1	0.983	1.1 ± 0.1	1.1 ± 0.1	0.648
Platelet count (×10^3^/μL)	156.0 ± 50.7	187.1 ± 67.7	0.262	157.4 ± 49.8	177.4 ± 72.1	0.190
Prior TACE (*n* [%])	8 (27.6%)	49 (22.2%)	0.448	7 (28.0%)	32 (25.8%)	0.807
Prior RFA (*n* [%])	2 (6.9%)	25 (11.3%)	0.750	2 (8.0%)	11 (8.8%)	1.000
AFP (ng/mL)	191.9 ± 604.0	474.8 ± 2369.8	0.318	103.5 ± 298.4	298.3 ± 1061.8	0.128
Preoperative Tumor size (cm)	4.2 ± 2.8	3.3 ± 2.2	0.013	4.0 ± 2.5	3.2 ± 2.1	0.139

All variables are presented as the mean and standard deviation or n (%) of patients. UPC, unplanned conversion; LLR, laparoscopic liver resection; BMI, body mass index; HTN, hypertension; MELD, model for end-stage liver disease; ALT, alanine aminotransferase; AST, aspartate aminotransferase; TACE, transarterial chemoembolization; RFA, radiofrequency ablation; AFP, α-fetoprotein.

**Table 2:** Surgical and oncological outcomes in matched cohort.

Variables	UPC (*N* = 25)	LLR (*N* = 120)	*p* Value
**Operation type (*n* [%])**			0.369
Right hemihepatectomy	8 (32.0%)	14 (11.2%)	
Right anterior sectionectomy	0	1 (6.4%)	
Right posterior sectionectomy	3 (12.0%)	16 (13.3%)	
Segmentectomy	3 (12.0%)	35 (28.0%)	
Tumorectomy	11 (44.0%)	54 (43.2%)	
**Operative parameters**			
Operation time (min)	337.2 ± 203.1	302.7 ± 173.2	0.254
Estimated blood loss (mL)	3172.1 ± 4527.0	809.4 ± 1026.4	<0.001
Transfusion (*n* [%])	12 (48.0%)	31 (24.8%)	0.028
Pringle maneuver (*n* [%])	15 (62.8%)	65 (52.0%)	0.379
Pringle maneuver (min)	40.5 ± 2.5	40.9 ± 27.9	0.783
**Postoperative data**			
Hospital stays (days)	14.8 ± 18.3	8.9 ± 8.3	0.015
C-D complications (*n* [%])			0.042
IIIa	6 (24.0%)	13 (11.8%)	
IIIb	1 (4.0%)	4 (3.6%)	
IV	0	2 (1.8%)	
V	0	1 (0.9%)	
**Pathologic data**			
R0 resection rate (*n* [%])	23 (92.0%)	119 (95.2%)	0.621
Surgical margin (cm)	0.9 ± 1.2	0.6 ± 0.8	0.523
Microvascular invasion (*n* [%])	10 (40.0%)	61 (48.8%)	0.512
Serosal invasion (*n* [%])	7 (28.0%)	31 (24.8%)	0.802
Tumor stage			0.701
I	11 (44.0%)	60 (48.0%)	
II	11 (44.0%)	54 (43.2%)	
III	2 (8.0%)	5 (4.0%)	
IV	1 (4.0%)	2 (1.6%)	
Total necrosis	0	4 (3.2%)	

All variables are presented as the mean and standard deviation or *n* (%) of patients. UPC, unplanned conversion; LLR, laparoscopic liver resection; C-D, Clavien-Dindo.

**Table 3:** Uni- and multivariable logistic regression analysis of risk factors Associated with poor overall survival and recurrence-free survival.

	Overall Survival			Disease Free Survival		
	Univariate Analysis	Multivariate Analysis		Univariate Analysis	Multivariate Analysis	
Risk Factor	*p* Value	HR (95% CI)	*p* Value	*p* Value	HR (95% CI)	*p* Value
Age, ≥65 (years)	0.027			0.385		
Male,	0.420			0.776		
BMI < 18.5 kg/m^2^,	0.022	2.073 (1.055–4.072)	0.034	0.961		
Previous abdominal surgery	0.065					
Preop. TACE	0.578			0.384		
Preop. RFA	0.574			0.407		
Child-Pugh score B	0.503					
Albumin < 3.5 g/dL	0.016	3.497 (1.600–7.646)	0.002	0.172		
Platelet count < 100 (×10^3^/μL)	0.481			0.001	2.081 (1.161–3.730)	0.014
Cirrhosis	0.439			0.286		
Tumor size > 5 cm	0.024	0.943 (0.472–1.885)	0.868	0.849		
Operation type	0.706			0.027		
LLR					Reference	
UPC					2.203 (1.045–4.643)	0.038
Operation time > 300 min	0.012	2.840 (1.121–7.194)	0.028	0.325		
Intraoperative blood loss > 1000 mL	0.002	1.379 (0.510–3.726)	0.527	<0.001	2.194 (1.119–4.299)	0.022
Intraoperative transfusion	0.002	0.768 (0.275–2.146)	0.614	<0.001	1.353 (0.701–2.609)	0.368
Resection margin (R1)	0.666			0.062		
Microvascular invasion	<0.001	2.503 (1.022–6.798)	0.042	0.002	2.401 (1.344–4.288)	0.003
Serosal invasion	0.760			0.873		
pT						
1	Reference			Reference		
2	0.872	1.063 (0.108–10.464)	0.985	0.217		
3	0.289	2.528 (0.223–28.698)	0.454	0.517		
4	0.013	6.692 (1.119–14.698)	0.041	0.299		
Major complication (C-D ≥ III)	0.162			0.182		
Hospital stay (>7 days)	0.043	0.921 (0.329–2.573)	0.875	0.250		

HR, hazard ratio; 95% CI, 95% confidence interval; BMI, body mass index; Preop., preoperative; TACE, transarterial chemoembolization; RFA, radiofrequency ablation; UPC, unplanned conversion; LLR, laparoscopic liver resection; C-D, Clavien-Dindo.

**Table 4:** Uni- and multivariable logistic regression analysis of preoperative variables associated with conversion.

	Univariable Analysis		Multivariable Analysis	
	HR (95% CI)	*p*	HR (95% CI)	*p*
Age		0.358		
<65 years	Reference			
≥65 years	1.442 (0.660–3.151)			
Male sex	1.067 (0.431–2.640)	0.889		
BMI		0.328		
<25 kg/m^2^	Reference			
≥25 kg/m^2^	1.474 (0.677–3.210)			
Hypertension	1.087 (0.489–2.141)	0.838		
Diabetes mellitus	1.594 (0.679–3.741)	0.285		
Previous abdominal surgery	0.566 (0.214–1.472)	0.267		
Previous TACE	1.310 (0.547–3.138)	0.544		
Previous RFA	0.584 (0.131–2.604)	0.480		
Albumin		0.001		0.001
<3.5 g/dL	4.808 (1.923–12.022)		4.873 (1.904–12.474)	
≥3.5 g/dL	Reference			
Platelet count		0.037		
<100 (×10^3^/μL)	1.391 (1.002–5.072)			
≥100 (×10^3^/μL)	Reference			
Cirrhosis (Preoperative Imaging)	1.586 (0.700–3.595)	0.269		
Tumor size		0.240		
<3 cm	Reference			
≥3 cm	1.593 (0.732–3.468)		1.014 (0.230–2.679)	0.714

HR, hazard ratio; 95% CI, 95% confidence interval; BMI, body mass index; TACE, transarterial chemoembolization; RFA, radiofrequency ablation.

## Data Availability

The datasets used and analyzed during the current study are available from the corresponding author upon reasonable request.
